# Differential Grainy head binding correlates with variation in chromatin structure and gene expression in *Drosophila melanogaster*

**DOI:** 10.1186/s12864-022-09082-7

**Published:** 2022-12-27

**Authors:** Henry A. Ertl, Mark S. Hill, Patricia J. Wittkopp

**Affiliations:** 1 Department of Ecology and Evolutionary Biology, University of Michigan, Ann Arbor, MI 48109 USA; 2 Present address: Cancer Evolution and Genome Instability Laboratory, University College London Cancer Institute and The Francis Crick Institute, London, UK; 3 Department of Molecular, Cellular, and Developmental Biology, University of Michigan, Ann Arbor, MI 48109 USA

**Keywords:** Pioneer factor, Evolution, Transcription factor, Chromatin accessibility

## Abstract

Phenotypic evolution is often caused by variation in gene expression resulting from altered gene regulatory mechanisms. Genetic variation affecting chromatin remodeling has been identified as a potential source of variable gene expression; however, the roles of specific chromatin remodeling factors remain unclear. Here, we address this knowledge gap by examining the relationship between variation in gene expression, variation in chromatin structure, and variation in binding of the pioneer factor Grainy head between imaginal wing discs of two divergent strains of *Drosophila melanogaster* and their F_1_ hybrid. We find that (1) variation in Grainy head binding is mostly due to sequence changes that act in *cis* but are located outside of the canonical Grainy head binding motif, (2) variation in Grainy head binding correlates with changes in chromatin accessibility, and (3) this variation in chromatin accessibility, coupled with variation in Grainy head binding, correlates with variation in gene expression in some cases but not others. Interactions among these three molecular layers is complex, but these results suggest that genetic variation affecting the binding of pioneer factors contributes to variation in chromatin remodeling and the evolution of gene expression.

**Supplementary Information:**

The online version contains supplementary material available at 10.1186/s12864-022-09082-7.

## Background

Metazoan development is guided by gene regulatory mechanisms that differentially express the genome to construct diverse cell and tissue types. Given the central role of gene regulation in development, it is perhaps not surprising that many instances of morphological evolution have been attributed to gene expression variation resulting from altered gene regulatory mechanisms [1, 2]. In many of these cases, at least some of the causative changes responsible for altering gene expression have been mapped to *cis*-regulatory DNA sequences [3–5], which bind transcription factors (TF) and activate transcription. The ability of *cis*-regulatory sequences to recruit transcription factors, however, is dependent not only their sequence but also on structural features of the genomic region in which they exist. Much of the genome is wrapped around nucleosomes and packaged into chromatin, and the molecular mechanisms that control chromatin structure and access to *cis*-regulatory sequences can also contribute to differences in gene expression within and between species [6].

Pioneer factors are a class of TF that can bind nucleosome-bound DNA and make it accessible for subsequent TFs to bind a *cis*-regulatory region and activate transcription. The activation of *cis*-regulatory elements by pioneer factors is thought to occur in two steps: (1) *cis*-regulatory regions are “primed” when pioneer factors bind, destabilize, and evict nucleosomes, making the regions moderately accessible, then (2) the *cis*-regulatory regions transition into an “active” state that allows other TFs to bind and recruit transcriptional machinery, making the regions fully accessible [7]. In some cases, pioneer factors help recruit the TF activators to *cis-*regulatory regions [8, 9]. From this mechanistic model, it is important to note that variation in chromatin accessibility can result from binding variation in both pioneer *and* non-pioneer transcription factors, however the model suggests that the former would likely have larger effects on downstream processes. These distinct and critical roles for pioneer factors in facilitating the transition from chromatin remodeling to transcriptional activation suggests that evolutionary changes in their binding might be an important source of diversity in chromatin accessibility and/or gene expression.

Here, we test these ideas by comparing the binding of pioneer factor Grainy head, chromatin accessibility, and gene expression (measured as mRNA abundance) between imaginal wing discs of two divergent strains of *Drosophila melanogaster*. Grainy head is a well-conserved transcription factor essential for epithelial cell development in flies, nematodes, and mice [10–13]. In *D. melanogaster*, it has been shown to be a pioneer factor, necessary and sufficient for chromatin accessibility [14]. The same study showed that Grainy head is ubiquitously expressed throughout imaginal discs but that the *cis*-regulatory targets of Grainy head were not ubiquitously activated, suggesting that Grainy head “primes” *cis*-regulatory regions by making them accessible to other transcription factors but is not sufficient itself to activate transcription. This study reported a correlation between chromatin accessibility and variation in the Grainy head recognition motif among lines in the Drosophila Genetic Reference Panel (DGRP, [15]), but did not examine the impact of this variation on gene expression [14] In the current study, we use more distantly related strains of *D. melanogaster* to investigate how sequence variation propagates from recognition motif to pioneer factor binding to chromatin accessibility to gene expression. In addition, we compare each of these layers not only between strains but also between alleles in F_1_ hybrids, which allowed us to separate the *cis*- and *trans*-acting components of this variation at each level. Taken together, these data show the extent to which sequence variation affecting pioneer factor binding likely contributes to the evolution of gene expression in *Drosophila*.

## Methods

### Fly strains, rearing, and wing disc collections

The two *D. melanogaster* genotypes compared in this study were the North American zygotic hybrid rescue (Zhr) strain [16] and the Zimbabwean isofemale strain Z30 [17]. This Z30 strain and other strains from Zimbabwe are thought to be in the early stages of speciation from North American strains of *D. melanogaster* [17, 18], with an estimated divergence time of ~ 10,000 years [19]. Each of these strains were previously subjected to 10 generations of sibling pair matings to reduce genome-wide heterozygosity [16]. All flies were reared on cornmeal medium using a 16:8 light:dark cycle at 25 °C. For each genotype (Zhr, Z30, and F_1_ hybrid), 10 vials were set up with five virgin females and five males, with Zhr females mated with Z30 males to produce F_1_ hybrids. From these vials, wandering female third instar larvae were collected based on the absence of testes, and imaginal wing discs were dissected in cold 1x PBS, snap frozen in liquid nitrogen, and kept at − 80 °C until all samples were collected. For Cut&Run samples, imaginal wing discs were lightly fixed (0.1% methanol-free formaldehyde for 2 minutes at room temperature) and quenched (125 mM Glycine) before snap freezing. Enough wing discs (see below) were collected to prepare Cut&Run, ATAC-seq, and RNA-seq libraries from Zhr, Z30, and the F_1_ hybrid genotypes with three biological replicates each, plus negative controls for Cut&Run using Immunoglobulin G (IgG), resulting in 30 total samples (3 genotypes × 3 biological replicates × 3 datatypes + 3 Igg).

### Cut&run and library preparation

100 lightly fixed imaginal wing discs were used for each sample. For Cut&Run [20], the protocol provided with the Cell Signaling Cut&Run Kit (CAT: 86652S) was used with the following minor modifications and specifications: (1) 200uL (instead of 1 mL) of 1x Wash Buffer was used to dounce homogenize the wing discs to ensure efficient pelleting, (2) the provided spike-in DNA was added at 1:100 dilution, and (3) for each sample, we used 3uL of a Grainy head antibody that targets an epitope on the C-terminus of *Drosophila* Grh [21]. To construct Cut&Run libraries, the NEB Ultra II Kit was used with the following modifications as described in [22], to adapt the manufacturers protocols for Cut&Run library preps of transcription factors. The fragment distribution of each sample was visualized with BioAnalyzer to confirm the presence of ~ 200-250 bp peaks representing TF-bound regions. Libraries were sequenced on Novaseq S4 300 cycle at the University of Michigan.

### ATAC-seq library preparation

10 imaginal wing discs were used for each sample. Wing discs were first lysed by spinning down (800×g for 5 mins at 4 °C) and replacing the supernatant with 50uL lysis buffer (10 mM Tris 7.5, 10 mM NaCl, 3 mM MgCl2, 0.1% IGEPAL CA-630). Lysed cells were spun down (800×g for 5 mins at 4 °C) and supernatant replaced with the transposition mix (25uL 2x TD Buffer, 2.5uL Tn5 Transposase, 22.5uL H2O). The transposition mixture was gently pipetted to mix and put at 37 deg for 30mins. The transposition reaction was stopped by adding 10uL of cleanup buffer (900 mM NaCl, 300 mM EDTA), 4 uL 5% SDS, 4uL Prot. K (20 mg/mL) and incubated at 37 deg for 30 minutes. The DNA containing Tn5-ligated adapters was cleaned up with Ampure beads at a 1.8X ratio (122.4uL AMPure XP to 68uL DNA) and eluted with 21uL H2O.

Libraries were amplified in two rounds. For the first round, 20uL of the DNA containing Tn5-ligated adapters was combined with 2.5uL 25uM Nextera primer 1, 2.5uL 25uM Customized Nextera primer 2, and 25uL NEBNext Hi-Fi 2x PCR Master Mix, and thermal cycled 9x according to the manufacturer’s instructions. The amplified libraries were then size selected with Ampure beads (0.5x right, 1.8x left) and eluted into 21uL H2O. The size-selected libraries were amplified again under the same conditions except for only 7 cycles. Finally, size-selected, amplified libraries were cleaned with Ampure XP beads at 1.5x ratio and eluted with 20uL H2O. Visualizing the fragment distribution of each sample with BioAnalyzer showed the nucleosome periodicity indicative of successful ATAC-seq library preparation. Libraries were sequenced on Novaseq S4 300 cycle at the University of Michigan.

### RNA-seq library preparation

10 imaginal wing discs were used for each sample. RNA was extracted from wing discs using the Carbonprep Trizol/Phenol protocol and reagents from Life Magnetics. Briefly, wing discs were placed in 500uL of Trizol, homogenized with a motorized pestle, bound to carbon beads, washed, eluted in H2O. mRNA sequencing libraries were then prepared with the Illumina stranded mRNA prep kit according to the manufacturer’s instructions. Libraries were sequenced on Novaseq S4 300 cycle at the University of Michigan.

### Sequencing read processing

All reads were trimmed of adapters and quality was assessed with the trimgalore package [23]. Reads from each sample were aligned to a concatenated Zhr and Z30 fasta file using bowtie2-align, sorted and indexed using samtools-sort, and duplicates were removed with samtools-rmdup for the ATAC- and RNA-seq but not Cut&Run libraries [24, 25]. Next, allele-specific alignments were extracted with samtools-view by filtering for uniquely aligning reads with no mismatches. Read counts are summarized in Table S1.

### Cut&run and ATAC-seq peak calling

To identify genomic regions enriched for Cut&Run signal, we used the macs2-callpeak function [26], using Cut&Run experiments with a nonspecific rabbit Igg antibody as the negative control. For ATAC-seq, we used the HMMRATAC program with default parameters, which is specifically designed for ATAC-seq data [27]. For both Cut&Run and ATAC-seq peak calling pipelines, after first examining each replicate separately, we merged biological replicates to maximize our power to call peaks using the samtools merge function [24]. To create consensus peak sets for Zhr and Z30, the called peak files for each dataset were concatenated and then merged using the BEDtools merge function [28].

### Counting reads, coordinate conversion, and quality filters

Aligned reads overlapping exonic regions for RNA-seq and consensus peak sets for Cut&Run and ATAC-seq were counted for each sample using the BEDtools multicov function [28]. The genomic coordinates for the Zhr and Z30 samples were then converted to dm3 and then to dm6 coordinates using previously constructed liftOver chain files [29]. We refined the datasets by retaining only regions/genes with greater than 20 reads mapping in all biological replicates of at least one genotype and 99% correct allele-specific mapping in all samples **(**Fig. S1**)**. We then normalized the read counts across samples with a counts per million transformation.

### Empirical Bayes model

To identify differentially Grh-bound/accessible regions and differentially expressed genes between parental strains or between alleles with the F_1_ hybrid, we adopted a similar statistical approach to one previously used [30]. Briefly, we used the Integrated Nested Laplace Approximation (INLA) framework [31] to estimate the posterior distribution of the difference in accessibility/expression either between parental strains or hybrid alleles. Specifically, we fit a logistic regression using the R INLA package [31] with a binomial likelihood family and default ‘minimally informative’ priors. To determine whether Grh binding/accessibility/expression was significantly different between parents/hybrid alleles, we estimated a two-tailed posterior predictive *P*-value, indicating whether the posterior estimate was equivalent to zero. Finally, we used the p.adjust() function to correct for multiple hypothesis testing.

### Identifying SNVs, Grh motifs, and computing PWMs

To identify single nucleotide variants (SNVs), we aligned genomic DNA reads from Zhr and Z30 [29] to the other’s personalized genome using bowtie2 [25] and then called SNVs using gatk Halotypecaller --genotyping-mode DISCOVERY --output-mode EMIT_ALL SITES --standard-min-confidence-threshold-for-calling 30. SNVs present in both reciprocal directions were retained for analyses. To identify Grh motifs, the Grh PWM was downloaded from JASPAR [32] and Grh motif coordinates were identified using the MEME suite FIMO function [33] with the Grh PWM, the dm6 genome assembly, and a statistical threshold of 1e-4. We then overlapped SNVs with Grh motifs, created Zhr and Z30 specific Grh motif sequences and calculated the PWM score as well as the difference between that of Zhr and Z30. PWM scores were calculated in R by converting the position frequency matrix to a PWM and summing the individual base scores of a given sequence, as described in [34]. Briefly, the position frequency matrix was converted to a PWM using the following equation:

$${W}_{b,i}=\mathit{\log}2\frac{p\left(b,i\right)}{p(b)}$$

where *W*_*b*, *i*_= PWM value of base *b* in position *i*; *p*(*b*) = background probability of base *b*; and *p*(*b*, *i*) = $\frac{f_{b+i}+s(b)}{N+\sum s\left({b}^{\prime}\right).}$

where *b*^′^ *ϵ* {*A*, *C*, *T*, *G*}; *f*_*b*, *i*_ = counts of base *b* in position *i*; *N* = number of sites; *p*(*b*, *i*) = corrected probability of base *b* in position *i*; and *s*(*b*^′^) = psuedocount function.

### Pairing regions and genes

Grh-Cut&Run regions were paired with ATAC regions using the BEDtools function intersect -F 1 to enforce only pairs for which the Grh-Cut&Run regions overlapped 100% with ATAC regions. These region pairs were then paired with the closest expressed gene using the BEDtools closest function [28].

## Results

### Experimental overview

**Fig. 1:**
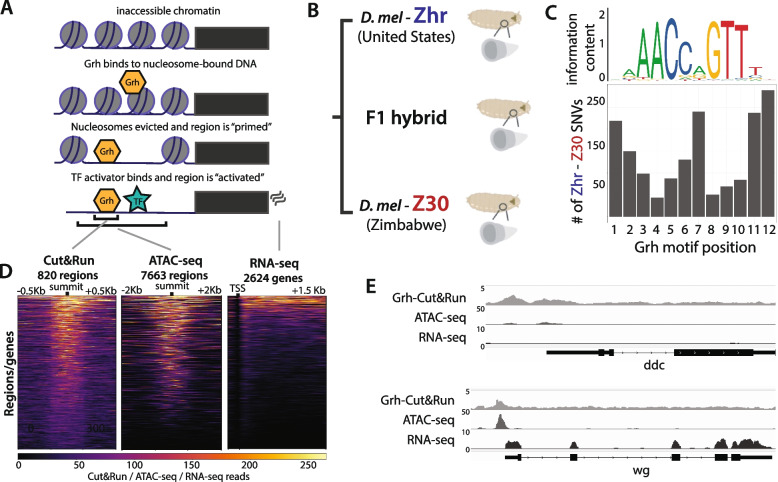
Experimental overview. **A** Schematic representation of Grh binding to nucleosome-bound DNA and making regions accessible for subsequent TF binding. **B** Third instar larval imaginal wing discs were dissected from the Zhr and Z30 strains (diverged ~ 10,000 years ago) and their F_1_ hybrid with three biological replicates per genotype. **C** Bar plot showing the number of single nucleotide variants at each Grh motif position across the Zhr/Z30 genome (bottom) aligned with the canonical Grh motif (top). **D** For one representative sample (Z30 replicate 1) heatmaps showing the read count across a window of each region and gene used for the analyses. **E** For the same sample shown in (D), screenshots from genome browser showing Cut&Run, ATAC-seq, and RNA-seq read counts at ddc (top) and wg (bottom)

To measure variation at multiple steps of gene regulation **(**Fig. 1A**)** and separate the *cis-* and *trans*-acting components of this variation, Grh binding (Cut&Run), chromatin accessibility (ATAC-seq), and gene expression (RNA-seq) data were collected from third instar larval imaginal wing discs of two *Drosophila melanogaster* strains*,* Zhr and Z30, and their F_1_ hybrids **(**Fig. 1B**)**. The Zhr and Z30 strains diverged ~ 10,000 years ago and have an average of 1.2% single nucleotide variants (SNVs) across the genome (Fig. S2A). At Grh motifs specifically, there are between 38 and 259 SNVs at each of the 12 positions in the motif, and the number of SNVs correlates with the position’s information content in the Grh binding motif **(**Fig. 1C**,** Fig. S2B), consistent with purifying selection preferentially filtering out variants that disrupt Grh binding.

To identify Grh bound regions of the genome, we called significant peaks from the Cut&Run data, of which the most highly enriched motif was the canonical Grh motif **(**Fig. 1C**, **top). We similarly called significant peaks from the ATAC-seq data, and then counted allele-specific reads that mapped to genes (RNA-seq) or peaks called in noncoding regions (ATAC-seq and Cut&Run) for each sample. After stringently filtering out genes/peaks with low read counts in all samples, evidence of allele-specific mapping bias, and/or Cut&Run regions without a Grh motif, we retained 820 Grh-bound regions, 7663 accessible regions, and 2624 expressed genes for analysis **(**Fig. 1D**,** Fig. S1). Read counts for each datatype were highly correlated across biological replicates with correlation coefficients ranging from 0.97 to 0.99. By comparison, correlation coefficients comparing data from Zhr to Z30 ranged from 0.91 to 0.97 **(**Fig. S3. To further assess the quality of our dataset, we compared our findings for specific loci (e.g., *ddc*, *wg*) **(**Fig. 1E**)** to those from prior work [35] and found that they were consistent with the earlier conclusion that Grh binding to promoters makes them accessible but does not necessarily activate transcription. Finally, to identify statistically significant differences in Grh binding, chromatin accessibility, and gene expression between the Zhr and Z30 genotypes, we used an empirical Bayes framework to estimate these parameters and then formally test for a difference between genotypes (Materials & Methods).

### Grainy head binding variation is primarily due to *cis*-acting changes outside of the Grainy head binding motif

**Fig. 2:**
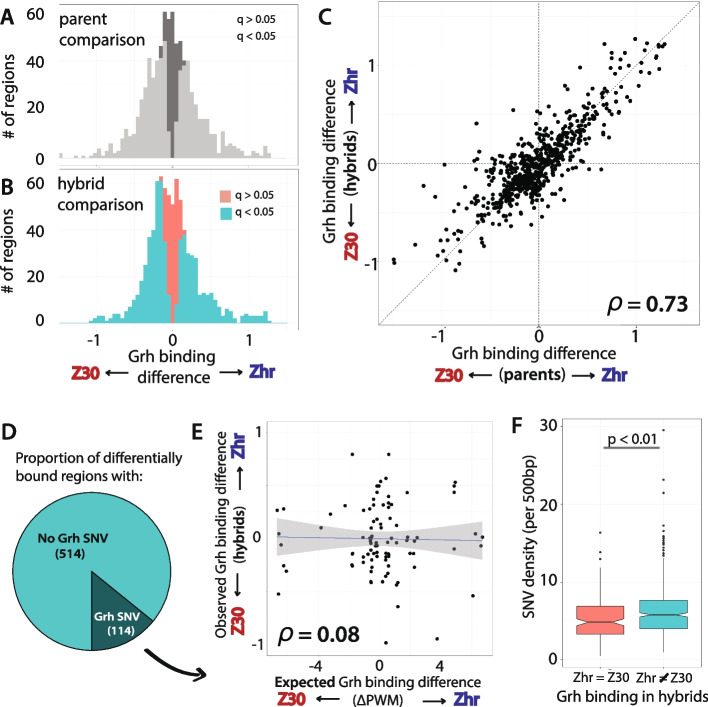
Grh binding variation is primarily due to *cis* changes outside the Grh binding motif. **A** Histogram of the estimated Grh binding variation between Zhr and Z30 parental strains. Bars are colored based on above (light gray) or below (dark gray) a q-value of 0.05 for the parental difference. **B** Histogram of the estimated Grh binding variation between Zhr and Z30 hybrid alleles. Bars are colored based on above (aqua) or below (salmon) a q-value of 0.05 for the hybrid allele difference. **C** Scatterplot contrasting the estimated Grh binding variation between parents (x-axis) versus the estimated Grh binding variation between hybrid alleles (y-axis). Spearman’s rho displayed in the bottom right corner. **D** Pie chart showing the ratio of variable Grh-bound regions with and without a SNV in the local Grh motif. **E** For Grh motifs with sequence variation, scatterplot contrasting the difference in position weight matrix score between Zhr and Z30 genotypes (x-axis) versus the empirically estimated Grh binding variation between hybrid alleles (y-axis). **F** The SNV density per 500 bp contrasted between Grh-bound regions with and without evidence for Grh binding variation (q < or > 0.05, respectively). Wilcoxon rank sum test: *p* < 0.01

Of the 820 Grh-bound regions identified, statistically significant differences in binding were observed for 651 regions between Zhr and Z30 **(**Fig. 2A**)**. Similarly, 628 regions showed significant differences in Grh binding between the Zhr and Z30 alleles in the F_1_ hybrids **(**Fig. 2B**)** (FDR < 0.05, Benjamini Hochberg correction). By comparing the difference in binding between the parents (Zhr and Z30) to that of the two alleles in the F_1_ hybrids for each region, we determined whether these differences in Grh binding were caused by genetic differences that act in *cis* or in *trans* [36]. Because the Zhr and Z30 *cis*-regulatory alleles are in a shared *trans-*regulatory environment in the F_1_ hybrid, variation between the hybrid alleles provides a direct measure the effects of *cis*-regulatory variation. The effects of *trans*-regulatory variation are then inferred from the difference between the Zhr and Z30 parental strains and the Zhr and Z30 hybrid alleles. We observed a strong correlation between the relative Grh binding to Zhr and Z30 alleles in the parents and F_1_ hybrids (Spearman’s rho = 0.73, *p*-value < 0.001), suggesting that most of the differences in Grh binding between the Zhr and Z30 strains are caused by *cis*-regulatory differences **(**Fig. 2C**)**.

We hypothesized that these differences in Grh binding attributable to *cis*-acting variation would be caused by changes in sequences matching the Grh binding motif. To test this hypothesis, we determined the number of differentially-bound regions with SNVs in the Grh binding motif. Surprisingly, only 18% of the regions with variable Grh binding contained a variable Grh binding motif **(**Fig. 2D**).** Moreover, even when there was a variable Grh motif in a region with variable Grh binding, the difference in predicted binding based on PWM scores and the empirically estimated Grh binding variation was not correlated **(**Spearman’s rho = 0.08, p-value = 0.54, Fig. 2E**).** These observations suggest that the source of the *cis*-acting variation causing differential Grh binding is likely located outside of the closest Grh binding motif. Because ​​TFs can collectively and collaboratively bind to *cis-*regulatory regions, we reasoned that sequence variation in adjacent binding sites for other factors might instead explain the observed variation in Grh binding. To explore this idea, we asked whether more variation was present in the sequence surrounding the Grh binding motif in regions that showed differential binding than in regions that did not. We found that the total number of SNVs was indeed greater in 500 bp regions with evidence of variable Grh binding than in regions where Grh binding was conserved between Zhr and Z30 **(**Fig. 2F**,** Wilcoxon rank sum test, *p* = 0.009).

### Grainy head binding variation correlates with changes in chromatin accessibility

**Fig. 3:**
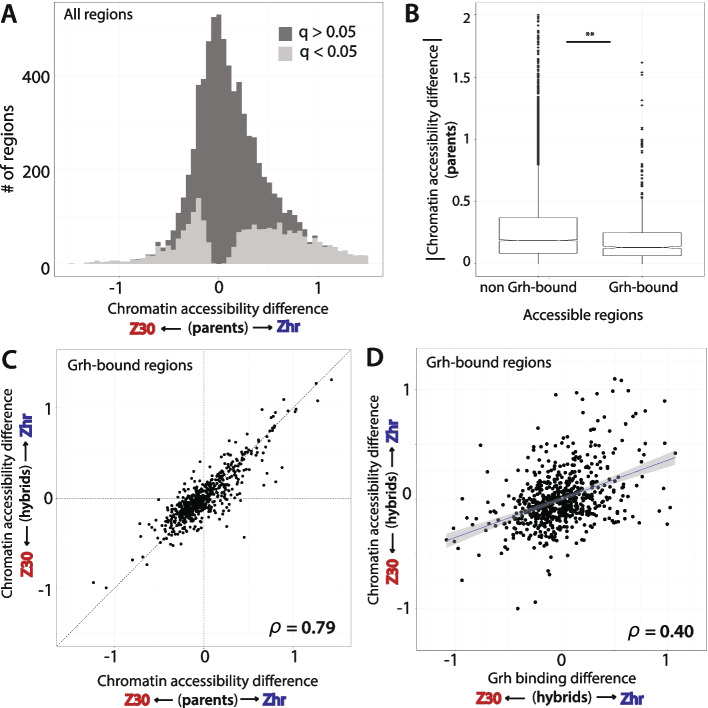
Variation in Grh binding correlates with changes in chromatin accessibility. **A** Histogram of the estimated chromatin accessibility variation of all regions between Zhr and Z30 parental strains. Bars are colored based on above (light gray) or below (dark gray) a q-value of 0.05 for the parental difference. **B** Boxplot contrasting the absolute value of the estimated parental difference in chromatin accessibility difference between accessible ATAC regions with and without Grh binding. Notches represent the 95% confidence interval around the median. **Wilcoxon rank sum test: *p* < 0.01 **C** For the 677 Grh-bound regions, scatterplot contrasting the estimated variation in chromatin accessibility between parents (x-axis) versus the estimated variation in chromatin accessibility between hybrid alleles (y-axis). Spearman’s rho displayed in the bottom right corner. **D** For the 677 Grh-bound regions, scatterplot contrasting the estimated variation in Grh binding between hybrid alleles (x-axis) versus the estimated variation in chromatin accessibility between hybrid alleles (y-axis). Spearman’s rho displayed in the bottom right corner. Line best fit to the data is shown, with 95% confidence intervals in shaded gray around the line

Because Grh is a pioneer factor, differences in Grh binding are expected to alter chromatin structure. To test this hypothesis, we used data from ATAC-seq in combination with the Cut& Run data described above to examine the relationship between Grh binding and chromatin accessibility. Overall, we found 4337 of 7663 regions with evidence of differential chromatin accessibility between the Zhr and Z30 strains (Fig. 3A), with the length of each accessible region ranging from 264 to 7353 bp (mean = 4007 bp). Of the 7663 total accessible regions, 677 overlapped with the 820 regions identified as Grh-bound in the Cut&Run data**.** Interestingly, chromatin accessibility is more conserved for the 677 Grh-bound regions than regions without evidence of Grh binding **(**Fig. 3B**)**. As with Grh binding, comparing differences in chromatin acessiblity at Grh-bound regions between the parental strains and the strain-specific alleles in F_1_ hybrids showed a strong correlation, suggesting that *cis*-regulatory variation was also primarily responsible for differences in chromatin accessibility **(**Fig. 3C, Spearman’s rho = 0.79, *p* < 0.001**)**. A similar correlation, albeit weaker, was seen when considering all (not just Grh-bound) accessible regions of the genome **(**Fig. S4**,** Spearman’s rho = 0.73, *p* < 0.001). This result is consistent with prior work also finding that local *cis*-regulatory changes primarily drive chromatin acessiblity variation [7]. To eliminate the impact of *trans*-regulatory differences between strains, we focused on comparing Grh-binding and chromatin accessibility between the Zhr and Z30 alleles in the F_1_ hybrids. For the 677 regions with evidence of both Grh binding and accessible chromatin, we found that differences in Grh binding were moderately correlated with differences in chromatin accessibility variation **(**Fig. 3D**,** Spearman’s rho: 0.40, *p* < 0.001**)**, suggesting that variation in Grh binding explains some, but not all, differential chromatin accessibility in these regions.

### Variation in chromatin accessibility at Grainy head-bound regions is moderately correlated with gene expression variation

**Fig. 4:**
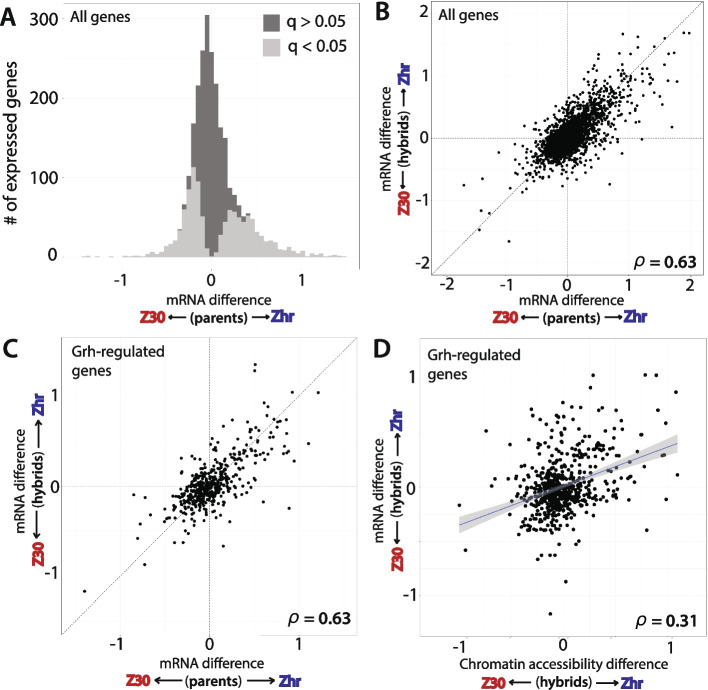
Variation in chromatin accessibility at Grh-bound regions is moderately correlated with gene expression variation. **A** Histogram of the estimated chromatin accessibility of all regions variation between Zhr and Z30 parental strains. Bars are colored based on above (light gray) or below (dark gray) a q-value of 0.05 for the parental difference. **B** For all genes, a scatterplot contrasting the estimated variation in gene expression between parents (x-axis) versus the estimated variation in gene expression between hybrid alleles (y-axis). Spearman’s rho displayed in the bottom right corner. **C** Same as B, but only for genes classified as Grh-regulated. **D** For Grh-regulated genes, scatterplot contrasting the estimated variation in chromatin accessibility between hybrid alleles (x-axis) versus the estimated variation in gene expression between hybrid alleles (y-axis). Spearman’s rho displayed in the bottom right corner. Line best fit to the data is shown, with 95% confidence intervals in shaded gray around the line

To understand whether and how variation in Grh binding propagates to chromatin accessibility and ultimately to gene expression, we used RNA-seq data to determine whether variation in chromatin remodeling was likely to affect levels of gene expression. First, we estimated mRNA differences of all 2624 expressed genes for parents and hybrid alleles and found that 1) 1138 genes show evidence of differential gene expression between Zhr and Z30 parental strains **(**Fig. 4A**)** and 2) most of this variation is due to *cis*-acting differences **(**Spearman’s rho: 0.632, p < 0.001, Fig. 4B**).** This contribution of *cis*-regulatory variation is much greater than that reported previously between these two strains of *D. melanogaster* using RNA extracted from whole adult flies [16, 37] and likely reflects the more focused tissue specific expression analyzed here. Next, we selected only the genes that were closest to the set of 677 Grh-bound (Fig. S5) accessible regions**,** again compared variation between parents and hybrid alleles, and found that the correlation for the Grh-regulated genes is nearly identical to that of all genes **(**Spearman’s rho: 0.631, p < 0.001, Fig. 4C**).** Consistent with this observation, we also found no evidence that the ratio between parental and hybrid allele differences (i.e., the mode of divergence: *cis* or *trans*) could be explained by Grh-binding (Anova, F_1,2844_ = 1.22, *P* = 0.27), suggesting that the relative roles of *cis-* versus *trans-*acting differences on gene expression are similar for genes with and without evidence of Grh-binding. Finally, we compared variation in chromatin accessibility at Grh-bound regions to that of gene expression and found a significant but weak correlation (Spearman’s rho: 0.31, p < 0.001) **(**Fig. 4D**)**. The relationship between variation in chromatin accessibility and gene expression at *all* accessible regions, however, was even weaker **(**Spearman’s rho: 0.25, p < 0.001, Fig. S6), suggesting that regions bound by Grh are more likely to show consistent variation in chromatin accessibility and gene expression.

### The relationship between Grainy head binding, chromatin accessibility, and gene expression variation

**Fig. 5:**
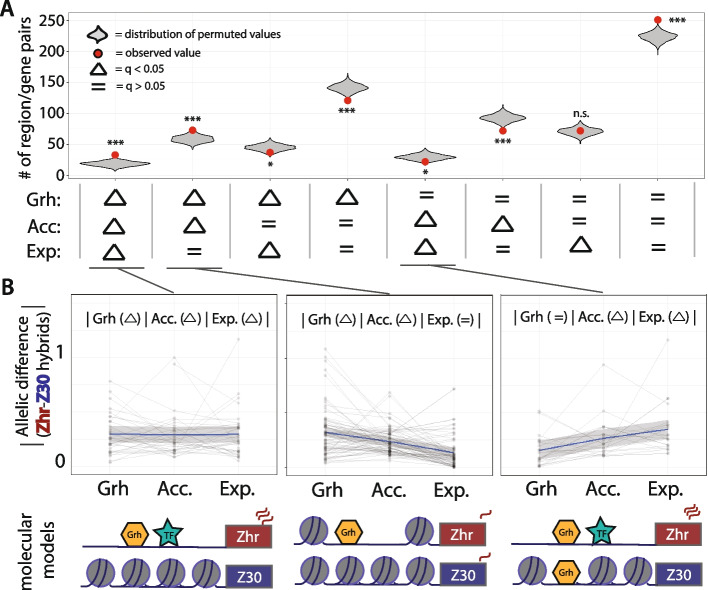
The relationship between Grh binding, chromatin accessibility, and gene expression variation. **A** Region/gene pairs were grouped based on evidence of a hybrid allelic difference (q < 0.05) for the indicated combination of data types (below plot). Evidence for and no evidence for a difference between the hybrid alleles is indicated by triangles and equal signs, respectively. For each group, the observed number of region/gene pairs is plotted as a red point, and the gray violin plots represent the distribution of counts obtained from 1000 permutations in which the rows (representing region/gene pairs) were shuffled for each datatype. From these permuted distributions, we calculated an empirical *p* value: *** < 0.001, * *p* < 0.05, ***, and n.s. = non-significant. **B** For three groups of interest, spaghetti plots of the absolute value of the estimated gene expression difference between hybrid alleles for Grh binding (Grh), chromatin accessibility (Acc.), and gene expression (Exp.). Loess lines (blue) were fit for each group to summarize the trends. For the molecular models, greater Grh binding, chromatin accessibility, and gene expression in Zhr is solely for the purposes of example; in some cases, the Z30 allele is expressed more strongly than the Zhr allele

The ultimate goal of this work was to try to connect DNA sequence variation to variation in binding of a pioneer factor (Grh) to variation in chromatin accessibility and variation in gene expression. To examine these relationships, we took a permutation approach to formally test the contribution of region/gene pairs where variation both does *and* does not propagate across mechanistic layers. More specifically, we grouped region/gene pairs based on the evidence of variable alleles (q value < 0.05) for either Grh binding, chromatin accessibility, or gene expression, and then calculated an empirical *p*-value by comparing the observed number of region/gene pairs in each category to a null distribution of analogous counts calculated from 1000 iterations of independently shuffling the three datatypes relative to regions/genes to break any real biological associations **(**Fig. 5A**,** Fig. S7). We found that (1) when coupled with Grh binding variation, chromatin accessibility variation is more likely to propagate to gene expression, but (2) a significant amount of Grh binding and chromatin accessibility does not have a measurable effect on gene expression. This first point is supported by finding that region-gene pairs with variation at all three steps are observed more often than expected by chance (p-value < 0.001, Fig. 5B, left), whereas region-gene pairs with variation in chromatin accessibility and gene expression but not Grh binding are observed less often than expected by chance (p-value < 0.04, Fig. 5B, right). In fact, the true number of concordant cases might be even greater than observed because the stringent cutoffs used to control the false positive rate might have created false negatives that would further increase the number of concordant cases. The second point is supported by the finding that region-gene pairs with variation in Grh binding and chromatin accessibility but not gene expression are observed more often than expected by chance (p-value < 0.01, Fig. 5B, middle). Region-gene pairs with no evidence of variation in Grh binding, chromatin accessibility, or gene expression were also observed more often than expected by chance (p-value < 0.001, Fig. 5A**).** Importantly, these results are robust to alternative methods of analysis (Fig. S8). Taken together, these results indicate that variation in binding of the Grh pioneer factor can be an important contributor to gene expression variation, but exactly how and when it has these effects likely depends on region- or gene-specific characteristics.

## Discussion

To understand the molecular changes that can contribute to the evolution of gene expression, we measured the contribution of variation in chromatin remodeling by a pioneer factor to gene expression variation between two distantly related strains of *D. melanogaster*. Prior studies have examined the relationship between variation in pioneer factor binding and chromatin accessibility [14] or variation in chromatin accessibility and gene expression [38] using strains of *D. melanogaster* isolated from a single population; however, by capturing more genetic divergence and examining all three levels in parallel, we were able to determine how changes in one level propagate to the next. We find that variation in Grh binding is nearly always caused by variation in *cis*-acting sequences and can explain some differences in chromatin accessibility. Regions of the genome in which variation in Grh binding overlaps with variation in chromatin structure are adjacent to differentially expressed genes more often than expected by chance, supporting the hypothesis that genetic variation affecting Grh binding can contribute to variation in gene expression by altering chromatin structure.

Similar relationships have also been described for other pioneer factors [9, 39], but it is important to keep in mind that a correlation between variation in Grh binding and chromatin accessibility should not necessarily be interpreted as Grh binding variation *causing* chromatin accessibility variation. That is, variation in Grh binding could also be a consequence of genetic variation impacting binding of other factors that indirectly alter the ability of Grh to bind to chromatin. Moreover, variation in Grh binding does not always explain variation in chromatin accessibility and that variation in chromatin accessibility does not always translate to variation in gene expression, as was also observed in studies of variation among the DGRP lines of *D. melanogaster* [38]. It is likely that these other sources of variation are non-pioneer transcription factors, since transcription factor binding in general is a main determinant of chromatin accessibility [7]. Variation in chromatin accessibility at any given Grh-bound region might also be different in other tissues (e.g., eye-antennal disc) because of differences in the *trans*-regulatory environment that can cause different transcription factors to bind to these regions [14, 40]. Taken together, these results suggest that variation in chromatin accessibility is the likely result of binding variation from many different TFs, both pioneer and non-pioneer, which is consistent with recent work on the determination of chromatin accessibility [40].

## Conclusions

In conclusion, these results provide insight into how variation in pioneer factor binding might contribute to variation in gene expression. But perhaps unsurprisingly, the relationship between mechanistic layers is complex: (1) sequence variation in Grh motifs rarely explains variation in Grh binding, which is consistent with prior work on binding variation of other pioneer and non-pioneer TFs [39, 41]; (2) variation in Grh binding only partly explains the variation in chromatin accessibility, despite the disproportionate role of pioneer factors in shaping chromatin structure [9]; and (3) there is a significant amount of variation in Grh binding and chromatin accessibility that both does and does not propagate to gene expression, and it is unclear what determines these two outcomes. Similar conclusions have been found in other *Drosophila* tissues [38] as well as other organisms, such as mice [39]. Future work to resolve these complexities will be made possible by continued work to understand the relationship between pioneer factor binding, chromatin accessibility of *cis*-regulatory regions, and ultimately the gene expression output that contributes to metazoan development.

## Supplementary Information

**Additional file 1.**

## Data Availability

The datasets generated and/or analyzed during the current study are available in the SRA repository, https://www.ncbi.nlm.nih.gov/sra/PRJNA867376. Accession Number: PRJNA867376. Scripts and files used for analysis are available at github.com/WittkoppLab/Ertl_et_al_AS_genomics.
