# Supplementary material for: Differential Grainy head binding correlates with variation in chromatin structure and gene expression in Drosophila melanogaster
Source: BMC Genomics. 2022 Dec 27;23:854. doi: 10.1186/s12864-022-09082-7 (PMC9795675; doi:10.1186/s12864-022-09082-7)
Supplement: Supplementary file 1 — Additional file 1. [file 12864_2022_9082_MOESM1_ESM.pdf]

Cut&Run	Zhr_rep1	Zhr_rep2	Zhr_rep3	Zhr_Igg	Z30_rep1	Z30_rep2	Z30_rep3	Z30_Igg	ZhrZ30_hyb_rep1	ZhrZ30_hyb_rep2	ZhrZ30_hyb_rep3	ZhrZ30_hyb_Igg
Mapped read count	31830054	75615531	93243324	75001085	45920087	78478336	92561058	98707783	99746460	99078988	72178151	95548121
Allele-specific mapped read count	7940182	17399400	23145532	17822737	11489309	19672055	22748894	23955654	22094835	23911405	18382991	23012723

ATAC-seq	Zhr_rep1	Zhr_rep2	Zhr_rep3	Z30_rep1	Z30_rep2	Z30_rep3	ZhrZ30_hyb_rep1	ZhrZ30_hyb_rep2	ZhrZ30_hyb_rep3
Mapped read count	41903438	22393174	51948002	8311594	33764542	10640158	55598232	52938501	48886537
Allele-specific mapped read count	10399221	5580341	12633291	2088122	8466205	2677190	13400192	13992394	11940212

RNA-seq	Zhr_rep1	Zhr_rep2	Zhr_rep3	Z30_rep1	Z30_rep2	Z30_rep3	ZhrZ30_hyb_rep1	ZhrZ30_hyb_rep2	ZhrZ30_hyb_rep3
Mapped read count	13145637	11813983	11428956	10343528	14645782	20046810	17827944	18093794	14342427
Allele-specific mapped read count	2601992	2332104	2271024	2070192	2912481	4023901	3509078	3617289	2899109

Table S1. Mapped read counts for each sample before and after allele-specific filtering. For all samples of Cut&Run, ATAC-seq, and RNA-seq libraries, counts of reads aligned via Bowtie2 to either the Zhr or Z30 genome before and after allele-specific filtering.

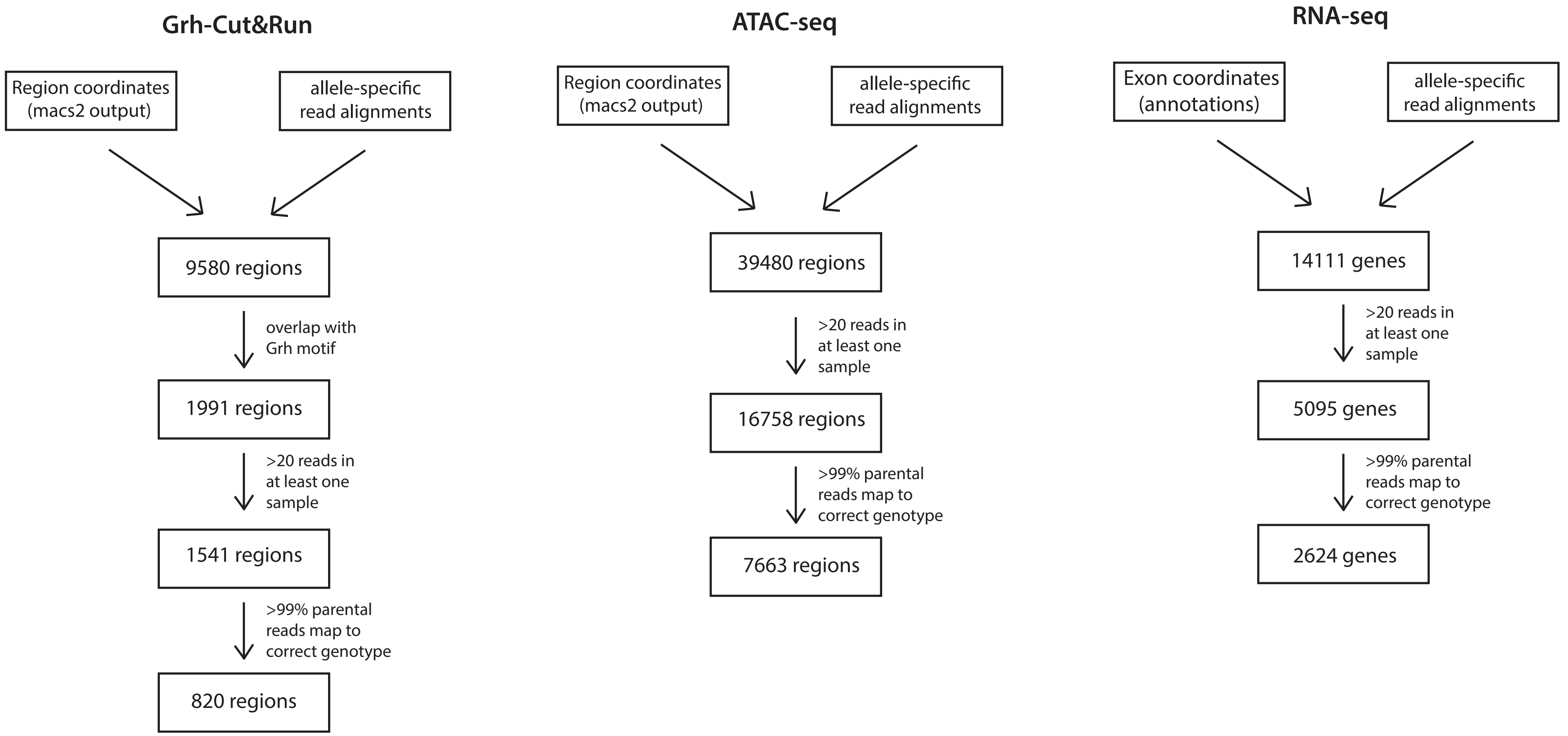

Figure S1. Flow chart for filtering regions and genes. A flowchart for each of the three datasets to show which filters were applied and how many regions/genes were filtered out at each step.

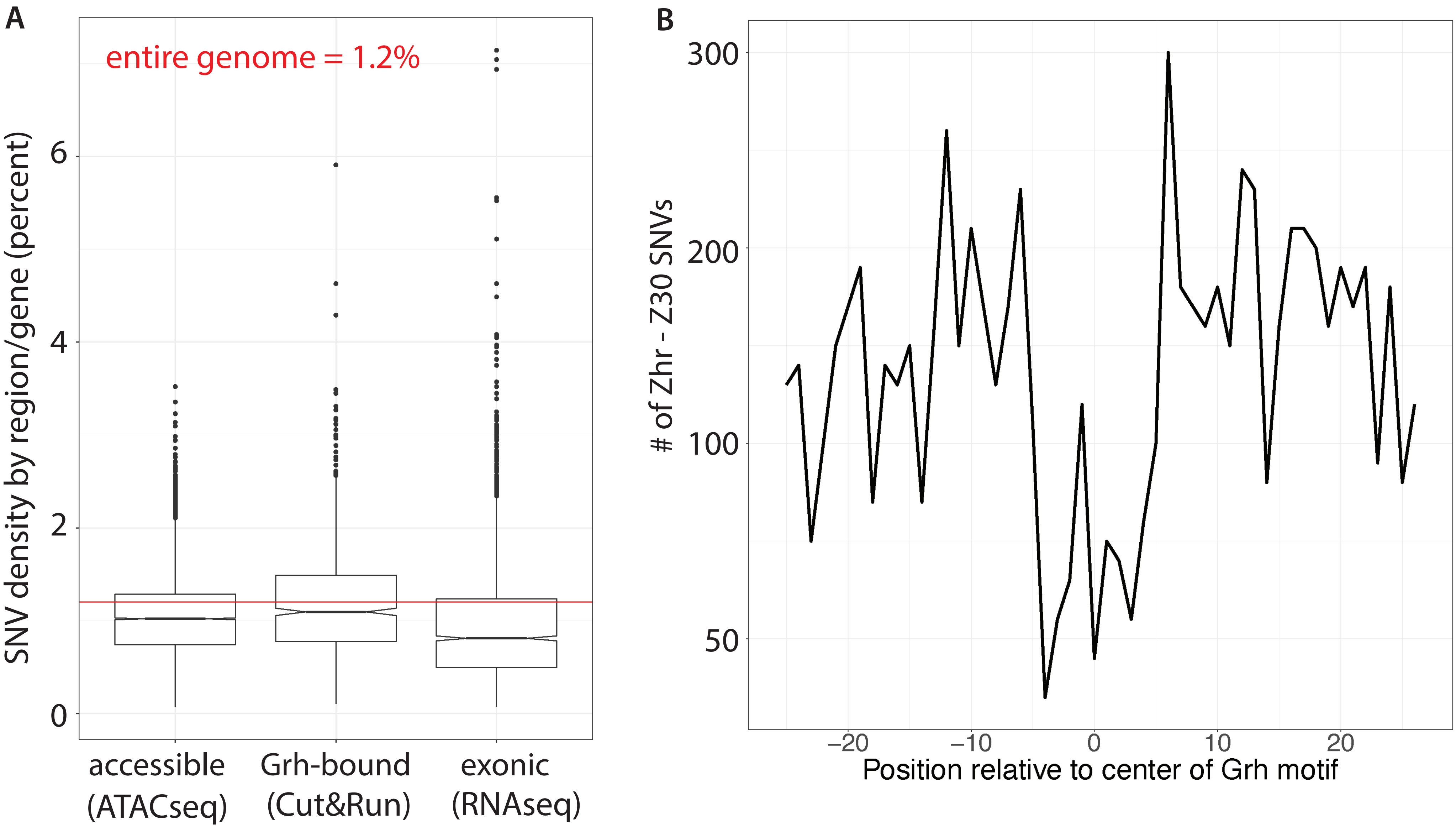

Figure S2. SNV characteristics across the genome and at Grh motifs. (A) Boxplots representing the distribution of SNV densities of each region (ATAC-seq and Cut&Run) or gene (RNA-seq) used for analyses. Notches represent the 95% confidence interval around the median. The red line represents the genome-wide percentage of SNVs. (B) Lineplot showing the number of SNVs at each Grh motif position (same as Figure 1B), as well as the surrounding 30bp for each focal Grh motif.

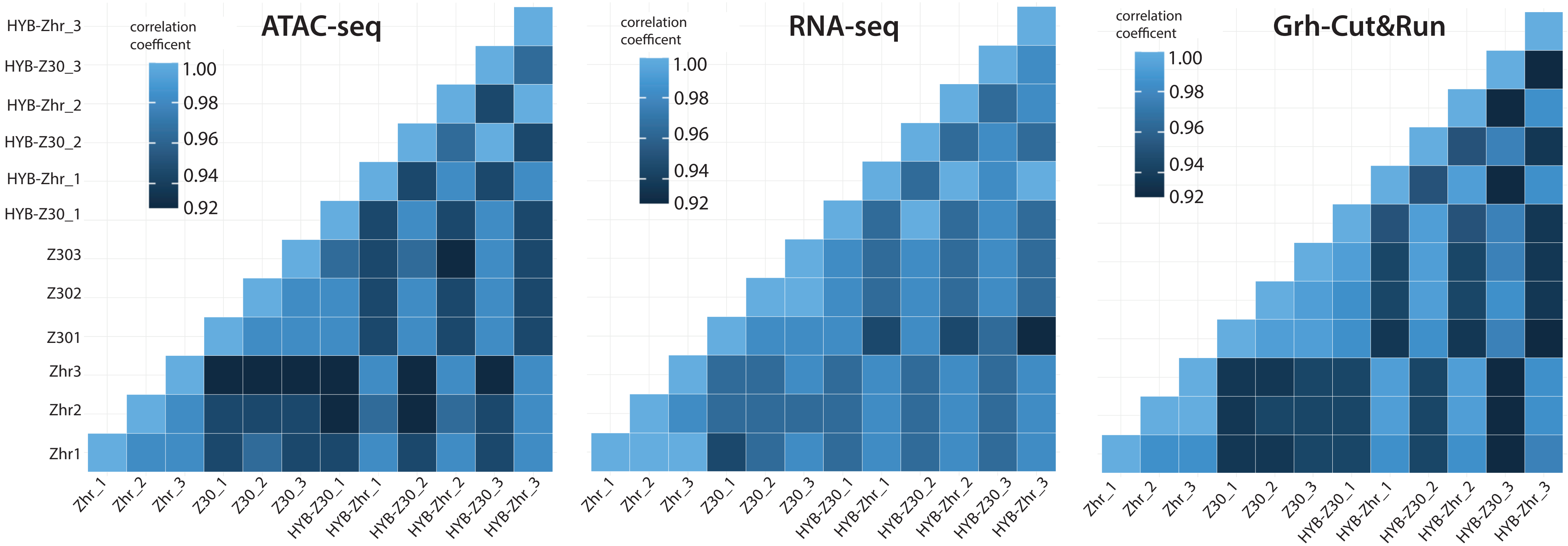

Figure S3. Correlation coefficients between samples. Pairwise Pearson's correlation coefficients between samples are represented as heatmaps for ATAC-seq (left), RNA-seq (middle), and Cut&Run (right). The values used for each sample are final, allele-specific CPM-transformed counts. The correlation coefficient value corresponds to the key, with lower values being blacker and higher values being bluer.

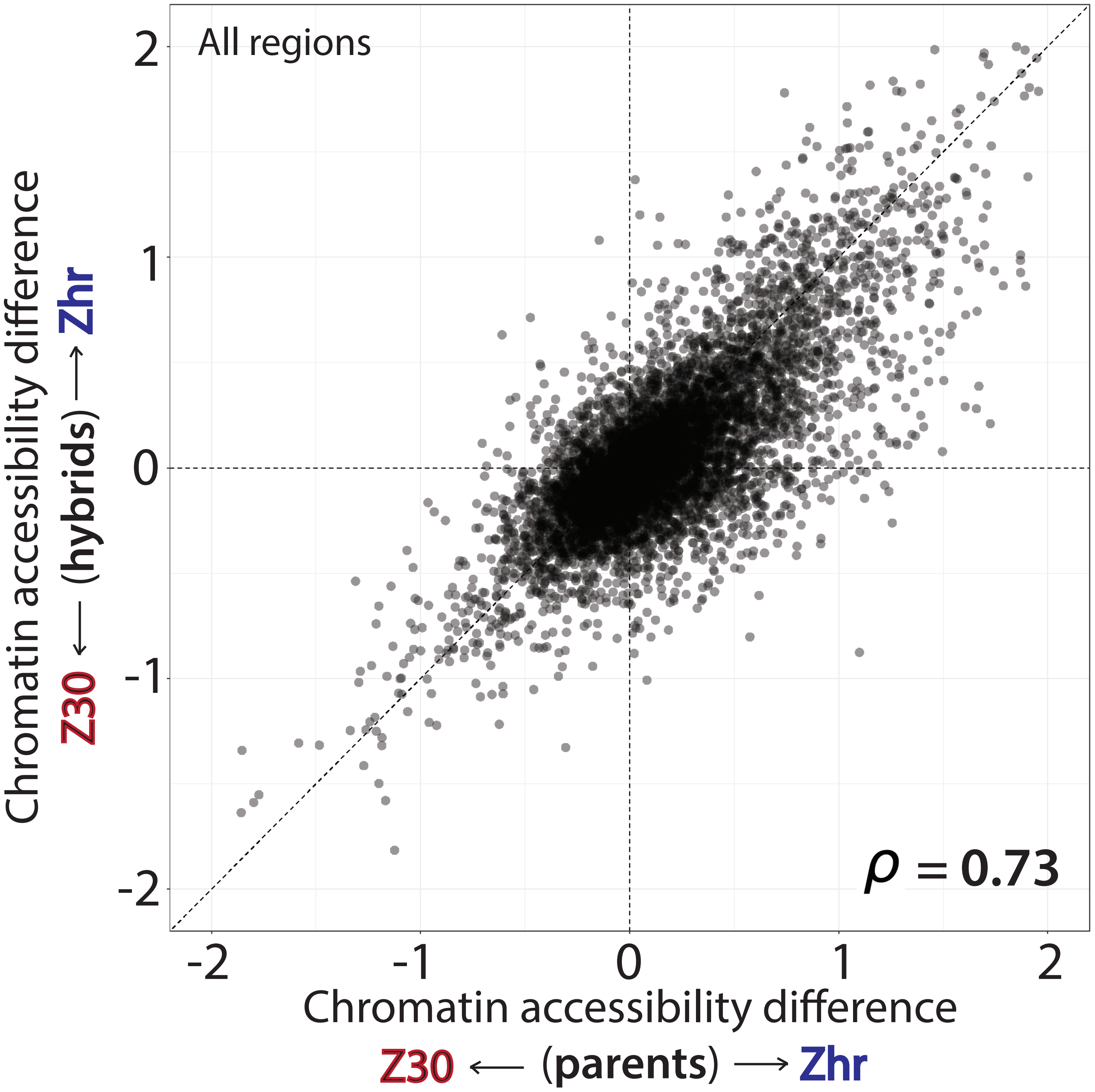

Figure S4. Chromatin accessibility features at Grh-bound regions. The same as Figure 4B but for all ATAC regions, instead of only those bound by Grh.

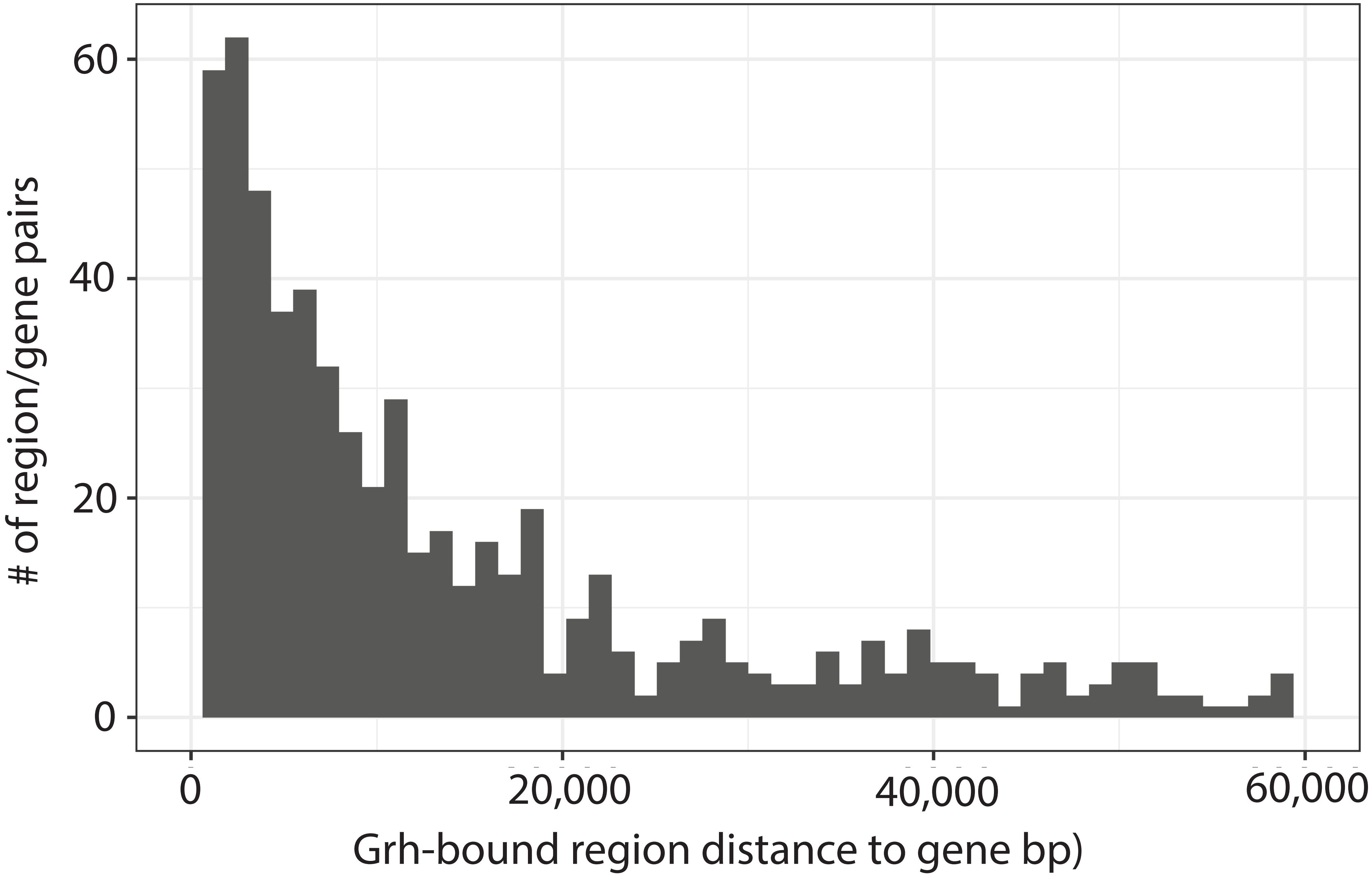

Figure S5. Distribution of distances between matched Grh-bound, accessible regions and expressed genes. For each pair, the distance (x-axis) was calculated by taking the absolute difference between the middle of the region and the transcription start site of the gene.

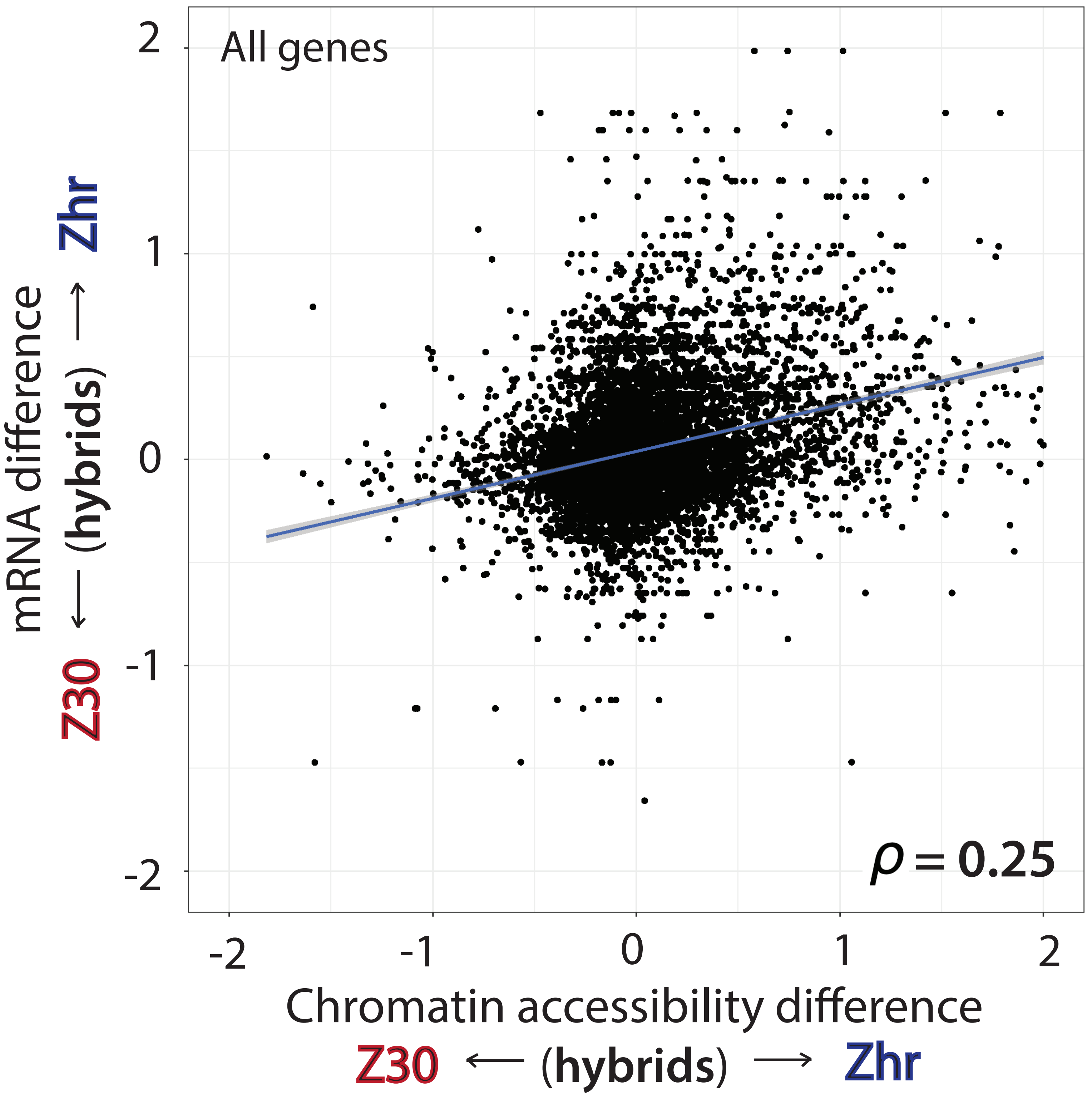

Figure S6. The correlation between variation in chromatin accessibility and gene expression for all genes. For all genes, a scatterplot contrasting the estimated variation in chromatin accessibility between hybrid alleles (x-axis) versus the estimated variation in gene expression between hybrid alleles (y-axis).

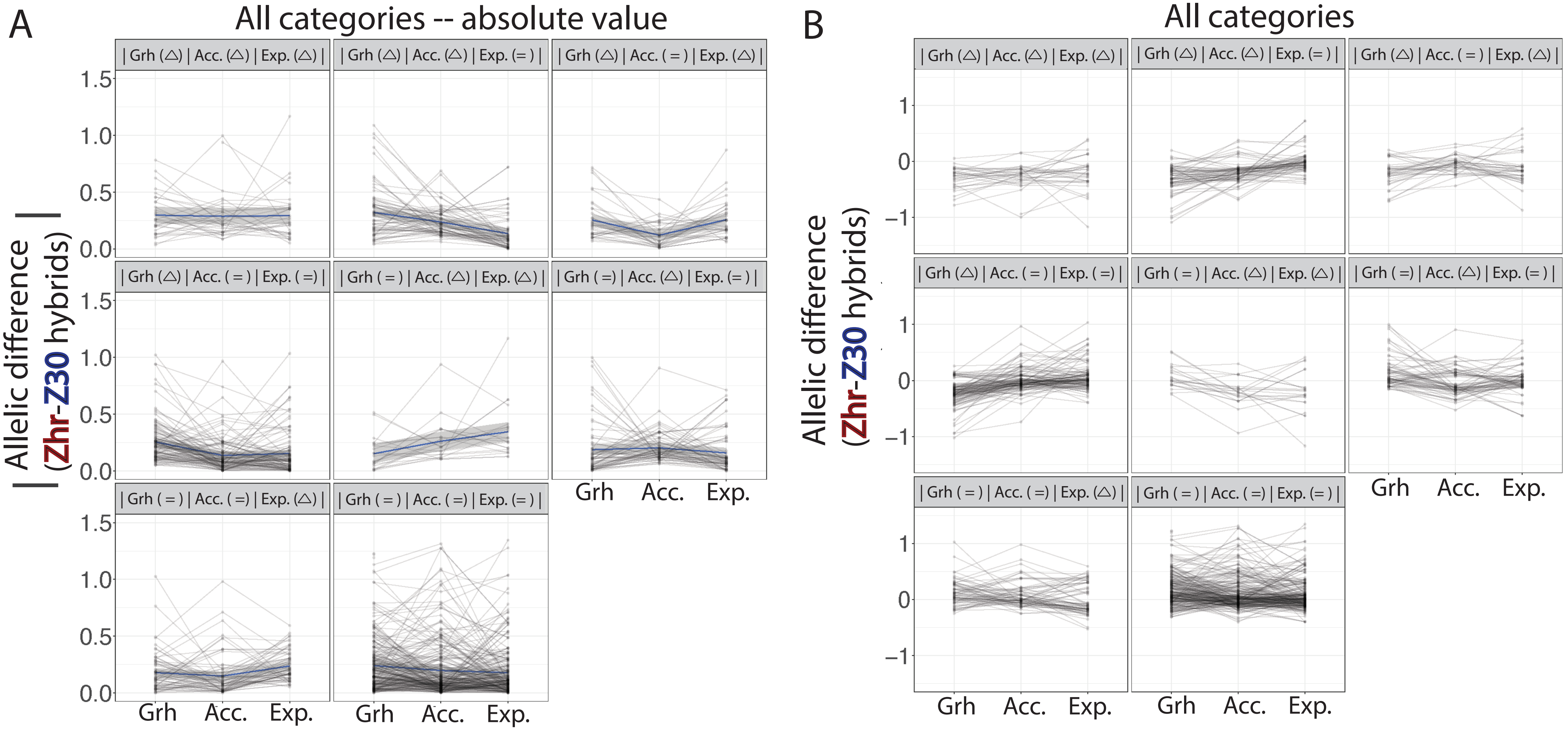

Figure S7. The relationship between variation in Grh binding, chromatin accessibility and gene expression for all gene/region pairs. For all region/gene categories, spaghetti plots of the (A) absolute value and (B) directional value of the estimated gene expression difference between hybrid alleles for Grh binding (Grh), chromatin accessibility (Acc.), and gene expression (Exp.). Loess lines (blue) were fit for each group to summarize the trends.

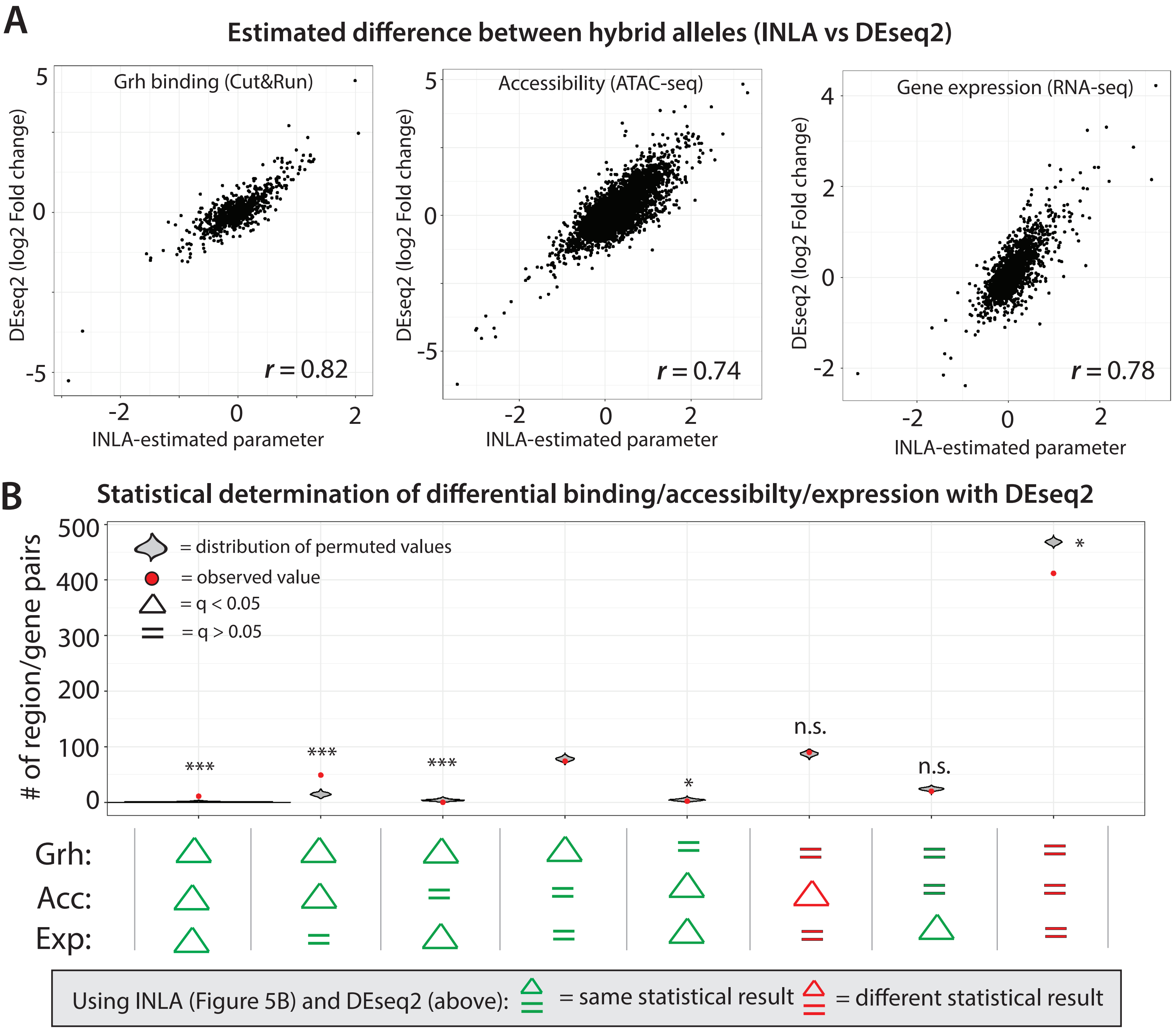

Figure S8. Differential Grh binding, chromatin accessibilty, and gene expression analyses repeated using DEseq2. Using DEseq2 with default parameters: (A) Scatterplots contrasting the estimated difference between hybrid alleles with DEseq2 (y-axis) versus that from INLA (x-axis), as used in the main text. Pearson correlation coefficients are provided in the bottom right for each datatype; (B) Permutation tests (as explained in the main text and Figure 5B) using the adjusted p-value output from DEseq2. The agreement/disagreement for significance and directionality between INLA and DEseq2 is indicated below (green = agreement; red = disagreement). ***: $q < 0.01$, *: $q < 0.05$, n.s. = not significant/$q > 0.05$.
